# Stability-Enhanced Ternary Solid Dispersions of Glyburide: Effect of Preparation Method on Physicochemical Properties

**DOI:** 10.1155/2023/2641153

**Published:** 2023-05-11

**Authors:** Leila Barghi, Afshin Vekalati, Azin Jahangiri

**Affiliations:** ^1^Department of Pharmaceutics, School of Pharmacy, Urmia University of Medical Sciences, Urmia, Iran; ^2^School of Pharmacy, Urmia University of Medical Sciences, Urmia, Iran

## Abstract

**Introduction:**

Limited aqueous solubility and subsequent poor absorption and low bioavailability are the main challenges in oral drug delivery. Solid dispersion is a widely used formulation strategy to overcome this problem. Despite their efficiency, drug crystallization tendency and poor physical stability limited their commercial use. To overcome this defect, ternary solid dispersions of glyburide: sodium lauryl sulfate (SLS) and polyethylene glycol 4000 (PEG), were developed using the fusion (F) and solvent evaporation (SE) techniques and subsequently evaluated and compared.

**Materials and Methods:**

Physicochemical and dissolution properties of the prepared ternary solid dispersions were evaluated using differential scanning calorimetry (DSC), infrared spectroscopy (FTIR), and dissolution test. Flow properties were also assessed using Carr's index and Hausner's ratio. The physical stability of the formulations was evaluated initially and after 12 months by comparing dissolution properties.

**Results:**

Formulations prepared by both methods similarly showed significant improvements in dissolution efficiency and mean dissolution time compared to the pure drug. However, formulations that were prepared by SE showed a greater dissolution rate during the initial phase of dissolution. Also, after a 12-month follow-up, no significant change was observed in the mentioned parameters. The results of the infrared spectroscopy indicated that there was no chemical interaction between the drug and the polymer. The absence of endotherms related to the pure drug from thermograms of the prepared formulations could be indicative of reduced crystallinity or the gradual dissolving of the drug in the molten polymer. Moreover, formulations prepared by the SE technique revealed superior flowability and compressibility in comparison with the pure drug and physical mixture (ANOVA, *P*  <  0.05).

**Conclusion:**

Efficient ternary solid dispersions of glyburide were successfully prepared by F and SE methods. Solid dispersions prepared by SE, in addition to increasing the dissolution properties and the possibility of improving the bioavailability of the drug, showed acceptable long-term physical stability with remarkably improved flowability and compressibility features.

## 1. Introduction

Type II diabetes is a global health priority and the most common form of diabetes [1]. Glyburide, a second-generation hypoglycemic sulfonylurea, is one of the most commonly prescribed oral hypoglycemic agents in the treatment of type II non-insulin-dependent diabetes mellitus [2]. The oral route of drug administration is the most common and preferred method of administration because of its convenience and ease of ingestion; however, it can be a difficult and inefficient method of administration for many drugs, mainly because of its low solubility [3]. Poor solubility and subsequent dissolution limited drug absorption, resulting in poor and variable bioavailability. It is noteworthy that the poor solubility often slows down the process of the formulation design as well as drug development and approval [4]. In this regard, the poor and inadequate bioavailability of glyburide can be improved by solubility and dissolution enhancement techniques. Several dissolution enhancement strategies have been considered to increase the solubility of drug substances, including solid dispersions, salt formation, micronization, micellar solubilization, modification of the drug crystal form, coacervation, complexation, nanoformulations, and co-solvent addition. Improving the dissolution rate and subsequently the oral absorption and bioavailability will also result in the reduction of dose and dose-dependent side effects [5–7]. The development of solid dispersions appears to be one of the most promising approaches to improving the dissolution rate and bioavailability of poorly water-soluble drugs. Improved solubility in solid dispersions has been attributed to several factors, including reduced drug crystallinity, a complete conversion to an amorphous state, reduced particle size, and improved wettability and porosity [8–11]. In solid dispersion systems, drug substances can be dispersed as separate molecules, amorphous particles, or crystalline particles in an inert carrier or matrix (which can be in the crystalline or amorphous form) in the solid state [5]. Amorphous solid dispersions have excellent performance in enhancing drug release rate, but subsequent drug supersaturation causes drug precipitation and lower drug concentration, thereby adversely affecting drug bioavailability. It may also recrystallize from an amorphous state during the manufacturing process or storage. Third-generation solid dispersions, in which surfactants are added as carriers or additives, have shown significant improvement in overcoming the aforementioned problems such as precipitation and recrystallization, thus enabling high drug-loaded amorphous solid dispersions [5, 12–15]. Two techniques commonly used to prepare solid dispersions are the fusion process (F) and the solvent evaporation process (SE). First, the drug and carrier are melted together, cooled, ground, and sieved to reduce the particle size. The miscibility of the drug and carrier in the molten form is essential in this process. The application of the F technique is limited to heat-resistant substances; however, this method does not involve a solvent or many steps [16]. In the SE technique, the solubility of both drug and carrier(s) in a common solvent is necessary. This method is appropriate for thermosensitive substances; however, sometimes the existence of residual solvent may be problematic [17]. In our previous study, glyburide binary solid dispersions were prepared and characterized [18]. The physical stability of the prepared solid dispersions was assessed for 6 and 12 months. Based on the obtained results, after 12-month storage, a significant decrease was observed in the DE, MDT, and dissolution profiles of the samples compared to the freshly prepared samples and samples stored for 6 months. Since physical stability is an important quality feature of amorphous solid dispersions [19], this study was designed to investigate the efficiency of ternary solid dispersions and the manufacturing process concerning formulation performance and physical stability. Ternary solid dispersions were prepared using polyethylene glycol (PEG) as a hydrophilic carrier and sodium lauryl sulfate (SLS) as a surface active agent by F and SE methods. Moreover, the effect of the preparation method was examined on physicochemical properties, physical stability, dissolution efficiency, flowability, and compressibility of the prepared formulations after preparation and after 12-month storage. Regarding the literature review, there are only a few comparative studies on the efficiency of F and SE techniques in the preparation of solid dispersions. On the other hand, most studies are focused on the kind and percentage of drug and carrier(s).

## 2. Materials and Methods

### 2.1. Materials

Glyburide was purchased from Mahban Chemical Co. Ltd. (Iran), and PEG 4000, monobasic potassium phosphate, sodium lauryl sulfate (SLS), sodium hydroxide, and methanol were bought from Merck (Germany).

### 2.2. Preparation of Glyburide Ternary SDs and Physical Mixtures (PMs)

Ternary solid dispersions of glyburide were prepared using different ratios of PEG and SLS by F and SE methods. Respective physical mixtures (PMs) were also prepared (Table 1). In brief, in the F technique, a precise amount of sieved PEG was placed in a suitable beaker and heated on a hotplate to 65–70°C until the PEG was completely melted. Accurately weighed amounts of each of the sieved SLS and glyburide powders were then added to the molten vehicle with continuous stirring until a homogeneous mixture was obtained. After complete dispersion, the prepared solid dispersion was then slowly cooled to room temperature. After 24 hours, the residue was ground and sieved through a 250 *μ*m sieve (60 mesh size). For the preparation of ternary SDs by the SE technique, accurately weighed and sieved combinations of the drug: polymer and surfactant (in 1 : 3 : 1, 1 : 5 : 1, and 1 : 9 : 1 ratios) were dissolved in a minimum volume of methanol and stirred at room temperature (Heidolph, MR Hei-Tec, Germany). After complete dissolution, the solvent was removed under reduced pressure using a rotary evaporator (Heidolph, Germany) at 60°C and 50 rpm for 24 h to ensure complete removal of the organic solvents. The residues were then pulverized by mortar and pestle and passed through a 250 *μ*m sieve (mesh size 60). To prepare the relevant PMs, precise amounts of sieved powders of glyburide, SLS, and PEG (<250 *μ*m) were mixed with a spatula for 5 min. All powders prepared by all three methods were stored in sealed tubes in a desiccator at room temperature (20–25°C) for subsequent characterization and experiments.

### 2.3. Saturated Solubility

To evaluate the polymer and surfactant effect on glyburide solubility, an excess amount of drug was added to 10 mL of various media including water, phosphate buffer (pH 7.8), saturated aqueous solutions of polymer, polymer, and surfactant mixture in phosphate buffer media. All tubes were sealed and kept under magnetic stirring at 25 ± 0.5°C for 24 hours. The samples were then filtered through a membrane filter (0.45 *μ*m), and the filtrate was diluted appropriately and analyzed for glyburide content using Cecil UV/VIS spectrophotometer at 301 nm.

### 2.4. Dissolution Studies

Drug release profiles of pure glyburide, PM, and formulations prepared by both F and SE techniques were studied using USP standard dissolution apparatus II, paddle stirrer (Pharmatest, Germany). Pure glyburide, PMs, and formulations all equivalent to 10 mg glyburide (*n* = 3) were added to the dissolution medium (900 mL, phosphate buffer pH 7.8, and 100 rpm stirring speed at 37 ± 0.5°C). At predetermined time intervals (10, 20, 30, 45, 60, and 120 min), 3 mL samples were taken using a 0.45 *μ*m membrane filter, 3 mL of fresh buffer was replaced, and the glyburide content was determined at 301 nm using a Cecil UV/VIS spectrophotometer. Finally, the cumulative amount of released drug was calculated and plotted against time [20]. For further investigation, the dissolution efficiency (DE) and mean dissolution time (MDT) of the prepared formulations were assessed and compared. The results presented are expressed as the mean ± standard deviation of three independent experiments.

### 2.5. Physical Stability

To assess the physical stability of the prepared solid dispersions by various techniques, the prepared samples were stored for 12 months in a desiccator under ambient temperature and humidity. After 12 months, the dissolution profile, DE, and MDT were evaluated in the prepared formulations.

### 2.6. Fourier-Transform Infrared Spectroscopy (FTIR) Study

FTIR spectra of the drug, polymer, surfactant, prepared solid dispersions, and PMs were obtained using a potassium bromide (KBr) disk method with an FT-IR spectrophotometer (PerkinElmer, USA). The infrared scanning wavelength was 4000−400 cm^−1^, and the resolution was 2 cm^−1^.

### 2.7. Differential Scanning Calorimetry (DSC)

DSC measurements were performed on the prepared solid dispersions and PMs as well as the pure drug, surfactant, and polymer, using a PerkinElmer DSC differential scanning calorimeter. Samples were hermetically sealed in aluminum DSC pans and placed in the DSC cell. Thermograms were acquired from 25°C to 250°C at a heating rate of 10°C/min.

### 2.8. Flowability and Compressibility

The flowability and compressibility of the pure drug, the prepared solid dispersion, and PM were evaluated using the Hausner ratio and Carr index, respectively. To measure the bulk and tapped densities, samples were gently poured from a glass funnel to the exact 10 mL mark into a graduated cylinder, and the weight of the powder required was determined. The cylinder was then tapped from a height of 1 cm until the final volume stopped decreasing. The bulk densities (*ρb*) and tapped densities (*ρt*) of pure glyburide, PM, and solid dispersions were determined, and the values of *ρb* and *ρt* were used to calculate the Carr index and Hausner ratio [21].

(1) $$\begin{array}{c} Carr's\;index\;\left(CI\right)=\left[1-\left(\frac{\rho b}{\rho t}\right)\right]\times 100\text{,}\\ Hausner's\;ratio\;\left(HR\right)=\frac{\rho t}{\rho b}\text{.} \end{array}$$

### 2.9. Statistical Analysis

One-way ANOVA was used to compare data between groups, followed by least significant difference (LSD) post hoc tests for pairwise comparisons (IBM SPSS Statistics 22). Differences between the groups were considered significant at *P*  <  0.05. All data are expressed as mean ± standard error of the mean obtained from triplicate samples.

## 3. Results and Discussion

### 3.1. Saturation Solubility Studies

The main purpose of this study was to evaluate the possibility of improving the solubility of glyburide by preparing ternary solid dispersion using SLS as a surfactant and PEG as a carrier. Based on the Noyes–Whitney equation, improving the saturated solubility of a drug may increase its dissolution rate [22].  Table 2 represents the saturation solubility studies of glyburide in various media. The saturation solubility of glyburide was found to be 11.67 ± 1.38 and 21.19 ± 0.73 mcg/mL in water and phosphate buffer, respectively. An approximately 5-fold increase in glyburide solubility was observed in the presence of polymer, and this observation is probably related to the increased wetting and solubilizing effect of the carrier [23–25]. In the presence of both polymer and surfactant, the increase in glyburide solubility was about 73-fold. A significant increase in glyburide solubility was evident in the polymer and surfactant mixture solution. This may be attributed to micellar solubilization, which is an excellent method to enhance drug solubility in aqueous environments [26]. Moreover, the addition of surfactant to solid dispersion could result in increased drug-polymer miscibility as well as reduced recrystallization [27, 28].

### 3.2. Dissolution and Physical Stability Studies

**Figure 1:**
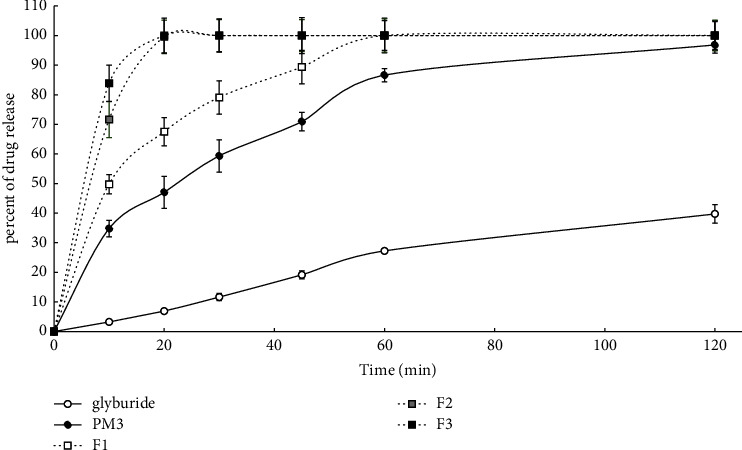
Comparison of dissolution profiles of glyburide and prepared formulations. Each point refers to mean ± SD (*n* = 3).

**Figure 2:**
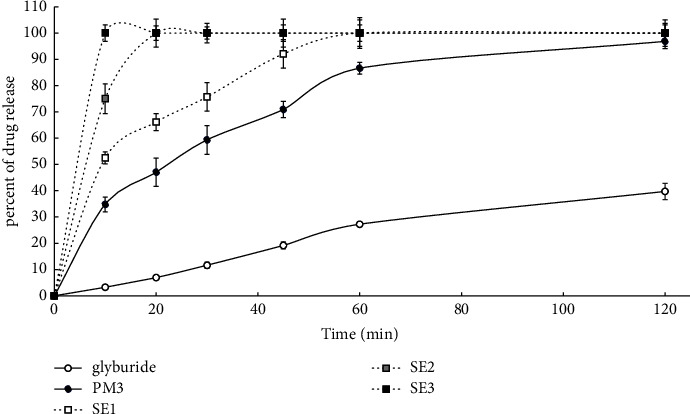
Comparison of dissolution profiles of glyburide and prepared formulations. Key: pure glyburide (○); PM3 (●); SE1 (□); SE2 (); SE3 (■). Each point refers to mean ± SD (*n* = 3).

In vitro dissolution profiles of the pure glyburide as well as the physical mixture and the prepared solid dispersions are shown in Figure 1 (solid dispersions prepared by F) and Figure 2 (solid dispersions prepared by SE). In the present study, dissolution data indicated that only around 27% of untreated crystalline glyburide dissolved at 60 min. It was also observed that the dissolution rate of PM increased (86% at 60 min), which might be related to increased wettability along with improved solubility caused by micellization [8]. In contrast to PM, both of the solid dispersions, prepared by F and SE, showed faster dissolution. However, solid dispersions prepared by SE displayed a better dissolution profile. In the prepared solid dispersions, the surfactant ratio was constant, but the polymer ratio was variable. The obtained results indicate that increasing the percentage of hydrophilic polymer resulted in improved wettability and subsequently enhanced surface hydrophilicity and dissolution performance [25]. So, 100% of the drug was released from SE3, SE2, and SE1 at 10, 20, and 60 min, respectively. The best result was obtained from SE3 and F3 (which contain the highest polymer ratio), and thus 100% of the drug was released from these formulations at 10 min and 20 min, respectively. Additionally, the partitioning of hydrophobic drug molecules into the micelle core and reduction in drug crystallinity have also been reported as another mechanism of increased dissolution rates in ternary solid dispersions [25, 29, 30]. In addition, surfactants applied to ternary solid dispersions prevent drug precipitation in aqueous media and improve wettability by reducing the interfacial energy barrier between the drug and dissolution medium [27].

As stated previously, to assess the physical stability of the prepared solid dispersions, samples were stored for 12 months in a desiccator under ambient temperature and humidity. After 12 months, the dissolution profile, DE, and MDT were evaluated and compared with the freshly prepared formulations. DE (percent) and MDT (min) of the pure glyburide and related formulations (right after and after 12-month storage) are shown in Table 3. Drug release percentages of solid dispersions prepared by F and SE methods (right after and after 12-month storage) are presented in Tables 4 and 5, respectively. Based on the obtained results, no significant change (*P*  <  0.05) was observed in DE, MDT, and dissolution profiles of freshly prepared samples compared to samples stored for 12 months. The use of surfactants in ternary solid dispersions not only improves the dissolution efficiency of poorly water-soluble drugs but also improves the physical stability. Due to the amphiphilic nature of the surfactant, it aids in improving the physical miscibility of hydrophobic drugs and reduces drug recrystallization [27, 31]. The highest and lowest values of DE belonged to SE3 (95.87%) and glyburide (22.88%), respectively. Among the solid dispersions prepared by the F method, F3 showed the best DE value (94.49%). DE and MDT values were observed in the following order for DE%: SE3 > F3 > SE2 > F2 > SE1 > F1 > PM3 > glyburide, and for MDT: SE3 < F3 < SE2 < F2 < SE1 < F1 < PM3 < glyburide. As can be seen, also in terms of DE and MDT, increasing the percentage of polymer led to increased dissolution performance in both methods.

According to Figures 1 and 2 and Tables 3–5, the dissolution performance of all solid dispersions was significantly improved, showing statistically significant differences (*P*  <  0.05) for dissolution profiles, DE, and MDT compared to glyburide and PM. Additionally, all formulations retained their dissolution performance after one year of storage. Other studies have also reported that surfactants in ternary solid dispersions showed obvious synergistic effects when combined with PVP, significantly reducing the crystal growth rate and improving physical stability [12].

### 3.3. DSC Study

**Figure 3:**
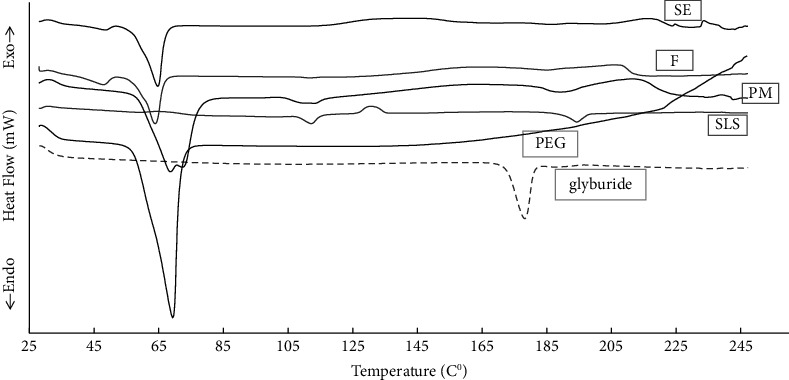
DSC thermograms of samples. Key: glyburide, PEG, and, SLS; physical mixture (PM), ternary solid dispersions prepared by fusion (F), and solvent evaporation (SE) techniques.

DSC thermograms of samples containing the pure drug, polymer (PEG), surfactant (SLS), physical mixtures (PMs), and solid dispersions prepared by melting (F) and solvent evaporation (SE) are shown in Figure 3. The DSC curves of glyburide and PEG showed sharp melting endotherms at 178°C and 69°C, respectively, indicating the crystalline structure of the samples. The SLS thermogram showed two endothermic peaks associated with dehydration and melting at 112°C and 195°C, respectively [32]. PM exhibited melting endotherms at 70°C, 112°C, and 190°C. The disappearance of the glyburide endothermic peak in the PM could be attributed to the gradual dissolution of the drug into the molten PEG during the DSC measurement, whereas in both solid dispersions prepared by F and SE methods, only PEG melting endotherm with complete disappearance of glyburide and SLS endothermic peaks was observed, suggesting the disappearance of the crystalline structure of SLS and glyburide or the gradual dissolving in the melted PEG during the DSC measurement [25, 33, 34]. The improved glyburide dissolution in the prepared solid dispersion compared to PM may be strongly associated with successful drug amorphization, as confirmed by DSC. Notably, the melting endotherms of PEG in the solid dispersion were detected at a slightly lower temperature (64°C) than that of the pure PEG (69°C). This decrease in the heat of fusion may be due to a decrease in the crystallinity of the PEG. Previous studies have suggested that the drug, mainly when mixed with the surfactant, has an amorphizing effect on the polymer, likely caused by hindering PEG recrystallization in solid-dispersed systems because of the existence of drug and surfactant in the mixture through the cooling or co-evaporation processes [34].

### 3.4. FTIR Study

**Figure 4:**
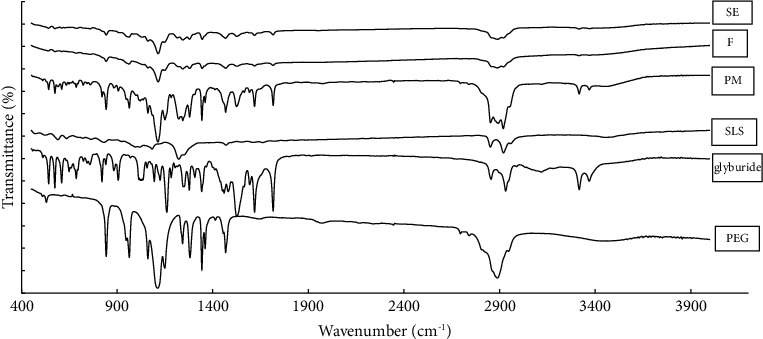
FTIR spectrum of samples. Key: glyburide, PEG, and SLS; physical mixture (PM), ternary solid dispersions prepared by fusion (F), and solvent evaporation (SE) techniques.

Fourier-transform infrared spectroscopy (FTIR) analysis was performed to identify potential drug-carrier interactions in the prepared formulations.  Figure 4 shows the FTIR spectra of the pure drug, polymer, and surfactant, along with the physical mixture and the prepared formulation. The FTIR spectrum of pure glyburide showed characteristic amide peaks at 3318 and 1715 cm^−1^, urea carbonyl stretching vibrations at 1618 and 1527 cm^−1^, and SO_2_ stretching vibrations at 1160 and 1342 cm^−1^ [35, 36]. The significant vibrations detected in the spectrum of PEG at 2890 cm^−1^, 1120 cm^−1^, and 3400 cm^−1^ were associated with C-H stretching, C-O stretching, and O-H stretching, respectively [37]. An SO_2_ stretch at 1222 cm^−1^ and a C-H stretch at 2920 cm^−1^ were detected in the SLS spectrum [38, 39]. The main peaks of the drug and carrier in the solid dispersion occurred at equal wavenumbers without band shifting and broadening; therefore, no interaction appeared to have occurred in the prepared formulations. These findings indicate that no chemical interactions occurred between the drug molecules and carriers during the manufacturing process of solid dispersions. Similar results have been reported for valdecoxib solid dispersion [40].

### 3.5. Flowability and Compressibility

Table 6 compares the flow properties of the untreated drug, the physical mixture, and the prepared formulations using the Hausner ratio and Carr index. Insufficient flowability can cause several obstacles during tableting, which undermine the processing time and the efficiency of die filling [41, 42]. The acquired results indicate that the untreated drug powder is cohesive with very poor flowability, while the prepared solid dispersions, those prepared using the SE method, represent improved flowability compared to the physical mixture and untreated drug powder (*P*  <  0.05). The improved powder flow might be attributed to the modified particle surface, shape, and size [41]. These results indicate that the method of solid dispersions preparation has had a significant effect on the improvement of the flow properties.

## 4. Conclusion

This work has shown that ternary solid dispersions of glyburide, prepared by both the F and SE techniques, presented improved physicochemical properties and physical stability. In the case of dissolution, MDT, DE%, and dissolution profile were improved in all prepared solid dispersions (both after preparation and after 12-month storage) compared to glyburide (*P*  < 0.05). The dissolution performance of solid dispersions prepared by SE was slightly better than that of F. Increased wettability and dispersibility along with molecular dispersion of the drug in the polymer matrix accompanied by a reduction in particle size and alteration of the surface properties of the drug particles might be responsible for the enhanced solubility and dissolution rate of glyburide in the prepared solid dispersions. FTIR spectroscopy showed no well-defined chemical interaction between glyburide, PEG, and SLS in the solid dispersion formulations. No endotherm related to glyburide was present in the DSC thermograms of the solid dispersions, suggesting complete miscibility of the glyburide in the melted carrier and/or disappearance of the drug crystalline structure. However, solid dispersions prepared by the SE method showed superior flowability and compressibility (*P*  < 0.05). Therefore, in this study, SE appears to be a suitable method for long-term improvement of the quality and performance of prepared glyburide ternary solid dispersions.

## Figures and Tables

**Table 1:** Theoretical composition, drug-to-polymer-to-surfactant ratio, and manufacturing process.

Drug : PEG : SLS	Manufacturing process	Formulation code
1 : 0 : 0	—	Glyburide
1 : 3 : 1	Fusion	F1
1 : 5 : 1	Fusion	F2
1 : 9 : 1	Fusion	F3
1 : 3 : 1	Solvent evaporation	SE1
1 : 5 : 1	Solvent evaporation	SE2
1 : 9 : 1	Solvent evaporation	SE3
1 : 3 : 1	Physical mixing	PM1
1 : 5 : 1	Physical mixing	PM2
1 : 9 : 1	Physical mixing	PM3

**Table 2:** Glyburide's saturated solubility.

Solubility medium	Glyburide solubility (mcg/mL) ± SD
Water	11.67 ± 1.38
Phosphate buffer (pH 7.8)	21.19 ± 0.73
Polymer solution in phosphate buffer	107.00 ± 10.31
Polymer and surfactant solution in phosphate buffer	1544.54 ± 21.85

**Table 3:** Dissolution efficiency (DE) and mean dissolution time (MDT) of the pure glyburide and related formulations (right after and after 12-month storage).

Formulation code	DE (%)	MDT (min)
Glyburide	22.88 ± 8.11	50.91 ± 16.82
PM3	73.13 ± 17.13	29.37 ± 9.25
F1	85.43 ± 16.75	17.48 ± 5.16
F1–12	82.65 ± 17.46	20.82 ± 5.16
F2	93.43 ± 13.38	7.88 ± 1.17
F2–12	92.41 ± 13.85	9.11 ± 5.16
F3	94.49 ± 11.22	6.61 ± 0.66
F3–12	93.37 ± 12.31	7.95 ± 5.16
SE1	85.53 ± 16.38	17.37 ± 5.35
SE1–12	83.44 ± 18.28	19.87 ± 5.16
SE2	93.75 ± 12.75	7.50 ± 1.02
SE2–12	92.75 ± 13.62	8.70 ± 5.16
SE3	95.87 ± 9.29	5.00 ± 00
SE3–12	95.51 ± 9.62	5.39 ± 5.16

Results are presented as mean ± standard deviation (*n* = 3).

**Table 4:** Investigation of drug release percentage from solid dispersions prepared by F (fresh and after 12-month storage).

Formulation code	F3		F2		F1	
Time	After 12 months	Fresh	After 12 months	Fresh	After 12 months	Fresh
10	76.37 ± 6.63	83.87 ± 6.08	68.19 ± 5.19	71.60 ± 6.08	44.32 ± 1.19	49.78 ± 3.24
20	94.10 ± 4.67	100 ± 5.85	90.69 ± 3.84	99.56 ± 5.64	61.37 ± 4.12	67.51 ± 4.74
30	100 ± 4.87	100 ± 5.64	100 ± 4.29	100 ± 5.40	73.64 ± 5.40	79.10 ± 5.64
45	100 ± 6.63	100 ± 6.08	100 ± 4.74	100 ± 5.40	83.87 ± 5.40	89.33 ± 5.64
60	100 ± 5.45	100 ± 5.01	100 ± 4.74	100 ± 5.64	98.19 ± 5.40	100 ± 5.85
120	100 ± 4.34	100 ± 4.67	100 ± 4.12	100 ± 5.19	100 ± 4.70	100 ± 5.01

Results are presented as mean ± standard deviation (*n* = 3).

**Table 5:** Investigation of drug release percentage from solid dispersions prepared by SE (fresh and after 12-month storage).

Formulation code	SE3		SE2		SE1	
Time	After 12 months	Fresh	After 12 months	Fresh	After 12 months	Fresh
10	96.15 ± 3.57	100 ± 3.15	69.55 ± 2.06	75.01 ± 5.64	45.0 ± 4.74	52.51 ± 2.25
20	100 ± 4.12	100 ± 2.74	93.42 ± 1.96	100 ± 5.30	55.91 ± 5.19	66.14 ± 3.24
30	100 ± 3.68	100 ± 2.34	100 ± 3.15	100 ± 3.68	72.28 ± 4.50	75.69 ± 5.40
45	100 ± 3.15	100 ± 5.30	100 ± 4.76	100 ± 3.15	90.01 ± 5.41	92.05 ± 5.40
60	100 ± 4.34	100 ± 5.01	100 ± 3.12	100 ± 3.15	100 ± 5.63	100 ± 5.85
120	100 ± 2.34	100 ± 3.68	100 ± 2.38	100 ± 3.12	100 ± 5.90	100 ± 5.01

Results are presented as mean ± standard deviation (*n* = 3).

**Table 6:** Flowability and compressibility index of the untreated drug as well as prepared formulations.

Formulation code	Compressibility index (%) (Carr's index)	Hausner ratio	Type of flow
Glyburide	37.94 ± 0.07	1.54 ± 0.09	Very poor
PM3	^*∗*^32.94 ± 0.08	1.49 ± 0.0	Very poor
F1	^*∗*^27.14 ± 0.18	^*∗*^1.37 ± 0.0	Poor
F2	^*∗*^30.06 ± 0.68	^*∗*^1.42 ± 0.0	Poor
F3	^*∗*^29.54 ± 0.65	1.41 ± 0.1	Poor
SE1	^*∗*^24.75 ± 0.09	^*∗*^1.33 ± 0.1	Passable
SE2	^*∗*^24.36 ± 0.17	^*∗*^1.32 ± 0.0	Passable
SE3	^*∗*^23.01 ± 0.09	^*∗*^1.32 ± 0.1	Passable

Results are presented as mean ± standard deviation (*n* = 3). ^*∗*^Statistically different at the significance level of 0.05 (compared to glyburide).

## Data Availability

The data used to support the findings of this study are included within this article.

## References

[1] Lau E., Carroll E., Callender L. (2019). Type 2 diabetes is associated with the accumulation of senescent T cells. *Clinical and Experimental Immunology*.

[2] Feldman J. M. (1985). Glyburide: a second‐generation sulfonylurea hypoglycemic agent: history, chemistry, metabolism, pharmacokinetics, clinical use and adverse effects. *Pharmacotherapy: The Journal of Human Pharmacology and Drug Therapy*.

[3] Singh N., Allawadi D., Singh S., Arora S. (2013). Techniques for bioavailability enhancement of BCS class II drugs: a review. *International Journal of Pharmaceutical and Chemical Sciences*.

[4] Sterren V. B., Zoppi A., Abraham-Miranda J., Longhi M. R. (2021). Enhanced dissolution profiles of glibenclamide with amino acids using a cogrinding method. *Materials Today Communications*.

[5] Vo C. L.-N., Park C., Lee B.-J. (2013). Current trends and future perspectives of solid dispersions containing poorly water-soluble drugs. *European Journal of Pharmaceutics and Biopharmaceutics*.

[6] Lateh L., Kaewnopparat N., Yuenyongsawad S., Panichayupakaranant P. (2022). Enhancing the water-solubility of curcuminoids-rich extract using a ternary inclusion complex system: preparation, characterization, and anti-cancer activity. *Food Chemistry*.

[7] Mallick S., Pattnaik S., Swain K., De P. K. (2007). Current perspectives of solubilization: potential for improved bioavailability. *Drug Development and Industrial Pharmacy*.

[8] Hu X.-Y., Lou H., Hageman M. J. (2018). Preparation of lapatinib ditosylate solid dispersions using solvent rotary evaporation and hot melt extrusion for solubility and dissolution enhancement. *International Journal of Pharmaceutics*.

[9] Jahangiri A., Barzegar-Jalali M., Garjani A. (2016). Evaluation of physicochemical properties and in vivo efficiency of atorvastatin calcium/ezetimibe solid dispersions. *European Journal of Pharmaceutical Sciences*.

[10] Butreddy A. (2022). Hydroxypropyl methylcellulose acetate succinate as an exceptional polymer for amorphous solid dispersion formulations: a review from bench to clinic. *European Journal of Pharmaceutics and Biopharmaceutics*.

[11] Dohrn S., Rawal P., Luebbert C. (2021). Predicting process design spaces for spray drying amorphous solid dispersions. *International Journal of Pharmaceutics X*.

[12] Kapourani A., Tzakri T., Valkanioti V., Kontogiannopoulos K. N., Barmpalexis P. (2021). Drug crystal growth in ternary amorphous solid dispersions: effect of surfactants and polymeric matrix-carriers. *International Journal of Pharmaceutics X*.

[13] Wang Y., Qin W., Liang Q., Zhou F., Yan C., Deng Y. (2021). The combination of hydroxypropylmethylcellulose acetate succinate and L-lysine into ternary amorphous solid dispersions of quercetin to enhance its dissolution. *Carbohydrate Polymer Technologies and Applications*.

[14] Giri B. R., Kwon J., Vo A. Q., Bhagurkar A. M., Bandari S., Kim D. W. (2021). Hot-melt extruded amorphous solid dispersion for solubility, stability, and bioavailability enhancement of telmisartan. *Pharmaceuticals*.

[15] Newman A., Zografi G. (2023). Considerations in the development of physically stable high drug load API- polymer amorphous solid dispersions in the glassy state. *Journal of Pharmaceutical Sciences*.

[16] Schönfeld B. V., Westedt U., Wagner K. G. (2021). Compression of amorphous solid dispersions prepared by hot-melt extrusion, spray drying and vacuum drum drying. *International Journal of Pharmaceutics X*.

[17] Eloy J. O., Marchetti J. M. (2014). Solid dispersions containing ursolic acid in Poloxamer 407 and PEG 6000: a comparative study of fusion and solvent methods. *Powder Technology*.

[18] Barghi L., Farajzadeh A., Jahangiri A. (2019). Preparation and evaluation of glibenclamide binary solid dispersions prepared by fusion and solvent-fusion method. *Biointerface Research in Applied Chemistry*.

[19] Bhujbal S. V., Mitra B., Jain U. (2021). Pharmaceutical amorphous solid dispersion: a review of manufacturing strategies. *Acta Pharmaceutica Sinica B*.

[20] Jahangiri A., Adibkia K., Asadpour-Zeynali K., Javadzadeh Y., Hamishehkar H., Barzegar-Jalali M. (2016). Application of multivariate calibration methods, in dissolution testing and simultaneous determination of atorvastatin and ezetimibe in their combined solid dosage form. *Pharmaceutical Sciences*.

[21] Kumavat S., Chaudhari Y., Borole P., Shenghani K., Badhe M. (2013). Enhancement of solubility and dissolution rate of curcumin by solid dispersion technique. *International Research Journal of Pharmacy*.

[22] Bolourchian N., Mahboobian M. M., Dadashzadeh S. (2013). The effect of PEG molecular weights on dissolution behavior of simvastatin in solid dispersions. *Iranian Journal of Pharmaceutical Research*.

[23] Ha E.-S., Ha D.-H., Kuk D.-H. (2017). Solubility of cilostazol in the presence of polyethylene glycol 4000, polyethylene glycol 6000, polyvinylpyrrolidone K30, and poly(1-vinylpyrrolidone-co-vinyl acetate) at different temperatures. *The Journal of Chemical Thermodynamics*.

[24] Patil M. P., Gaikwad N. J. (2009). Preparation and characterization of gliclazide-polyethylene glycol 4000 solid dispersions. *Acta Pharmaceutica*.

[25] Biswal S., Sahoo J., Murthy P. N., Giradkar R. P., Avari J. G. (2008). Enhancement of dissolution rate of gliclazide using solid dispersions with polyethylene glycol 6000. *AAPS PharmSciTech*.

[26] Alizadeh M. N., Shayanfar A., Jouyban A. (2018). Solubilization of drugs using sodium lauryl sulfate: experimental data and modeling. *Journal of Molecular Liquids*.

[27] Chaudhari S. P., Dugar R. P. (2017). Application of surfactants in solid dispersion technology for improving solubility of poorly water soluble drugs. *Journal of Drug Delivery Science and Technology*.

[28] Dave R. H., Patel A. D., Donahue E., Patel H. H. (2012). To evaluate the effect of addition of an anionic surfactant on solid dispersion using model drug indomethacin. *Drug Development and Industrial Pharmacy*.

[29] Afrasiabi Garekani H., Aftabi S. F., Nia F. F., Javidi M., Nokhodchi A., Sadeghi F. (2019). Synergistic effect of polyethylene glycol and superdisintegrant on dissolution rate enhancement of simvastatin in pellet formulation. *Pharmaceutical Development and Technology*.

[30] Shahba A. A., Tashish A. Y., Alanazi F. K., Kazi M. (2021). Combined self-nanoemulsifying and solid dispersion systems showed enhanced cinnarizine release in hypochlorhydria/achlorhydria dissolution model. *Pharmaceutics*.

[31] Alhijjaj M., Belton P., Qi S. (2017). A multi-technique characterization of the stability of surfactant containing solid dispersion based buccal patches prepared by hot melt injection moulding. *International Journal of Pharmaceutics*.

[32] Freire F., Aragão C., de Lima e Moura T., Raffin F. (2009). Compatibility study between chlorpropamide and excipients in their physical mixtures. *Journal of Thermal Analysis and Calorimetry*.

[33] Okonogi S., Puttipipatkhachorn S. (2006). Dissolution improvement of high drug-loaded solid dispersion. *AAPS PharmSciTech*.

[34] Mura P., Moyano J. R., González-Rodríguez M. L., Rabasco-Alvaréz A. M., Cirri M., Maestrelli F. (2005). Characterization and dissolution properties of ketoprofen in binary and ternary solid dispersions with polyethylene glycol and surfactants. *Drug Development and Industrial Pharmacy*.

[35] Raghavendra H., Kumar G. P. (2017). Development and evaluation of polymer-bound glibenclamide oral thin film. *J Bioequiv Availab*.

[36] Bahri-Najafi R., Tavakoli N., Senemar M., Peikanpour M. (2014). Preparation and pharmaceutical evaluation of glibenclamide slow release mucoadhesive buccal film. *Research in pharmaceutical sciences*.

[37] Gorajana A., Rajendran A., Yew L. M., Dua K. (2015). Preparation and characterization of cefuroxime axetil solid dispersions using hydrophilic carriers. *International journal of pharmaceutical investigation*.

[38] Barkvoll P., Embery G., Rølla G. (1988). Studies on the interaction between sodium lauryl sulfate and hydroxyapatite using Fourier transformed infrared spectroscopy. *Journal de Biologie Buccale*.

[39] Chakraborty S., Ghosh M., Chakraborti S. (2015). Biosurfactant produced from Actinomycetes nocardiopsis A17: characterization and its biological evaluation. *International Journal of Biological Macromolecules*.

[40] Shah J., Vasanti S., Anroop B., Vyas H. (2009). Enhancement of dissolution rate of valdecoxib by solid dispersions technique with PVP K 30 & PEG 4000: preparation and in vitro evaluation. *Journal of Inclusion Phenomena and Macrocyclic Chemistry*.

[41] Szabó E., Démuth B., Galata D. L. (2019). Continuous formulation approaches of amorphous solid dispersions: significance of powder flow properties and feeding performance. *Pharmaceutics*.

[42] Démuth B., Nagy Z. K., Balogh A. (2015). Downstream processing of polymer-based amorphous solid dispersions to generate tablet formulations. *International Journal of Pharmaceutics*.

